# Neuroprotective and Anti-Inflammatory Activities of Allyl Isothiocyanate through Attenuation of JNK/NF-κB/TNF-α Signaling

**DOI:** 10.3390/ijms18071423

**Published:** 2017-07-03

**Authors:** Lalita Subedi, Ramu Venkatesan, Sun Yeou Kim

**Affiliations:** Laboratory of Pharmacognosy, College of Pharmacy and Gachon Institute of Pharmaceutical Sciences, Gachon University, #191, Hambakmoero, Yeonsu-gu, Incheon 21936, Korea; subedilali@gmail.com (L.S.); vramamoorthysree@gmail.com (R.V.)

**Keywords:** allyl isothiocyanate, microglia, neuron, astrocyte, neuroinflammation, neuroprotection

## Abstract

Allyl isothiocyanate (AITC), present in *Wasabia japonica* (wasabi), is an aliphatic isothiocyanate derived from the precursor sinigrin, which is a glucosinolate present in vegetables of the Brassica family. Traditionally, it has been used to treat rheumatic arthralgia, blood circulation, and pain. This study focuses on its anti-apoptotic activity through the regulation of lipopolysaccharide (LPS)-induced neuroinflammation. Furthermore, we assessed its neuroprotective efficacy, which it achieves through the upregulation of nerve growth factor (NGF) production. Pretreatment with AITC significantly inhibited inducible nitric oxide synthase (iNOS) and cyclooxygenase-2 (COX-2) expression, decreased tumor necrosis factor-α (TNF-α), interleukin-6 (IL-6), prostaglandin E2 (PGE2), and nitric oxide (NO) production in activated microglia, and increased the nerve growth factor (NGF) and neurite outgrowth in neuroblastoma cells. AITC inhibited the nuclear factor (NF-κB-mediated transcription by modulating mitogen activated protein kinase (MAPK) signaling, particularly downregulating c-Jun N-terminal kinase (JNK) phosphorylation, which was followed by a reduction in the TNF-α expression in activated microglia. This promising effect of AITC in controlling JNK/NF-κB/TNF-α cross-linking maintains the *Bcl-2* gene family and protects neuroblastoma cells from activated microglia-induced toxicity. These findings provide novel insights into the anti-neuroinflammatory effects of AITC on microglial cells, which may have clinical significance in neurodegeneration.

## 1. Introduction

Neuroinflammation and neuronal cell death are the major causes of most neurological diseases [1]. Neurodegeneration is believed to be initiated by neuroinflammation [2]. The most common neurodegenerative and neuroinflammatory conditions are Alzheimer’s disease (AD) and Parkinson’s disease (PD) [3,4]. Injury and toxins, such as lipopolysaccharide (LPS), trigger the defense systems of the brain, which leads to neurodegeneration [5]. LPS induces an inflammatory cascade in microglia through an increased production and accumulation of cytotoxic and inflammatory mediators such as nitric oxide (NO), prostaglandin E2 (PGE-2), arachidonic acid, eicosanoids, and reactive oxygen species (ROS). Furthermore, it elevates the levels of proinflammatory cytokines, such as tumor necrosis factor-α (TNF-α), interleukin-6 (IL-6), and interleukin-1β (IL-1β), which are responsible for neuronal death [6,7]. Therefore, controlling microglial activation can be a therapeutic approach in the treatment of neuroinflammation as well as neurodegenerative conditions.

The use of natural products and functional foods as medicine (nutraceuticals) has considerably increased in the last two decades [8]. Natural compounds are useful in the treatment of various conditions, and often do not have the toxic side effects of synthetic compounds. Hence, many studies have focused on the consumption of healthy foods and medicinally effective herbs. Food components or phytochemicals we consume everyday can control the normal balance of the body without producing unwanted changes [9]. Hence, researchers are attempting to identify nutraceuticals that are effective against neurodegenerative diseases. In this study, we focused on allyl isothiocyanate (AITC), which is found in many vegetables of the Brassica family. AITC is an aliphatic isothiocyanate derived from the precursor sinigrin, which is a glucosinolate found in vegetables of the Brassica family, including Brussels sprout, cabbage, cauliflower, kale, mustard, horseradish, and wasabi [10].

*W. japonica* (wasabi) is a pungent spice popular in certain parts of Asia, including Japan and Korea, where it is used to prepare traditional foods such as sashimi and sushi. Its rhizome is used as a condiment in Japan [11]. Traditionally, it has been used to treat rheumatic arthralgia, as it promotes blood circulation and alleviates pain [12]. Several naturally occurring bioactive compounds, such as 6-(methylsulfonyl) hexyl isothiocyanate (6-MITC), AITC, and sinapic acid (SA), are present in wasabi. 6-MITC has anti-inflammatory, chemopreventive, and anti-melanoma activity [13]. AITC is one of the major isothiocyanates in cruciferous vegetables, including Brussels sprout, cabbage, cauliflower, kale, mustard, horseradish, and wasabi, which are widely consumed [14,15]. Originally, it had been stored as the precursor sinigrin [16]. Furthermore, it is used as a food additive and flavoring agent because of its strong smell and taste [17]. AITC has been shown to possess bioactivity, including antimicrobial, anti-gastric, immune-boosting, and antioxidant activities, in a variety of cells [18,19]. Previous studies reported that AITC has an excellent pharmacokinetic profile in rats and mice, revealing that more than 90% of bioavailability and almost 80% of the administered amount had been excreted. The lipophilicity of AITC, with its strong oral bioavailability and better excretion ability, made it an interesting probe for the treatment of various disease conditions, including cancer. Moreover, AITC has been found in the tissue distribution after oral administration, and was also detected in the brain [17]. These previous findings revealed that AITC can cross the blood–brain barrier. The anti-inflammatory effect of AITC in LPS-stimulated RAW cell (murine macrophages) has previously been reported [10]. Furthermore, Xiang et al. reported that isothiocyanate protected the blood–brain barrier from oxidative stress-induced damage [20]. However, it remains unclear whether AITC is involved in neuroinflammation and/or neuroprotection. Moreover, the anti-neuroinflammatory and neuroprotective effects of AITC on glial cells and neurons have not been investigated. Therefore, we examined the role of AITC in the control of neuroinflammation, and its neuroprotective effects in an in vitro system comprising microglia, neurons, and astrocytes.

## 2. Results

### 2.1. Effects of AITC on NO Production and iNOS, COX-2, and TLR4 Expression in LPS-Stimulated BV2 Cells

**Figure 1:**
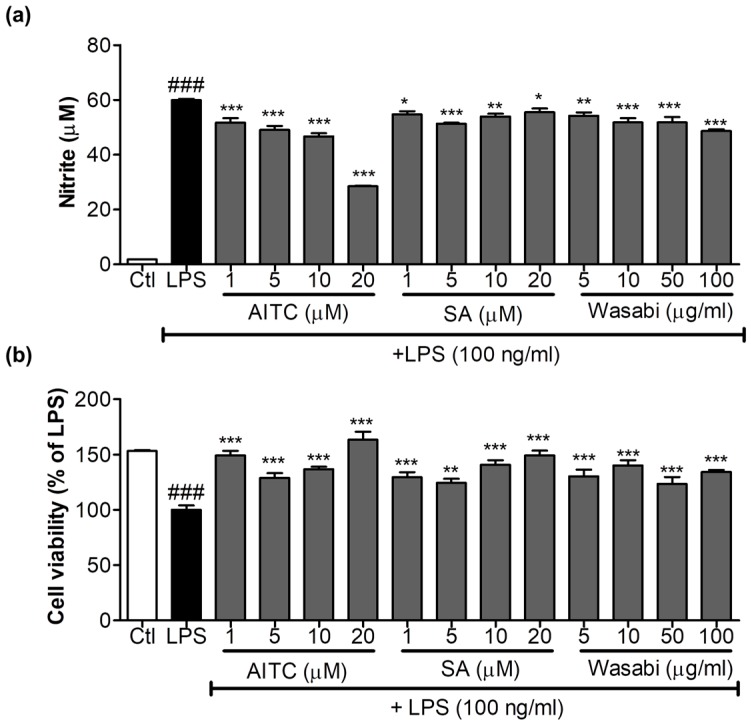
Effects of allyl isothiocyanate (AITC), sinapic acid (SA), and wasabi extract on nitric oxide (NO) production in lipopolysaccharide (LPS)-stimulated murine microglia (BV2) cells. BV2 cells were pretreated with 1, 5, 10, or 20 µM of AITC and SA and 5, 10, 50, and 100 µg/mL of wasabi extract for 30 min, and were stimulated with 100 ng/mL LPS for 24 h. Conditioned medium was used for nitrite measurement, and cells were used for the cell viability assay. (**a**) AITC and the wasabi extract inhibited LPS-induced NO production; (**b**) Cytotoxicity of compounds and extracts to BV2 microglia was determined using the MTT assay. All data are presented as the mean ± SEM of three independent experiments performed with *n* = 3. The LPS-treated group was considered as 100% for the cell viability assay. * *p* < 0.05, ** *p* < 0.01, and *** *p* < 0.001 vs. LPS-treated group; and ### *p* < 0.001 vs. untreated control group.

**Figure 2:**
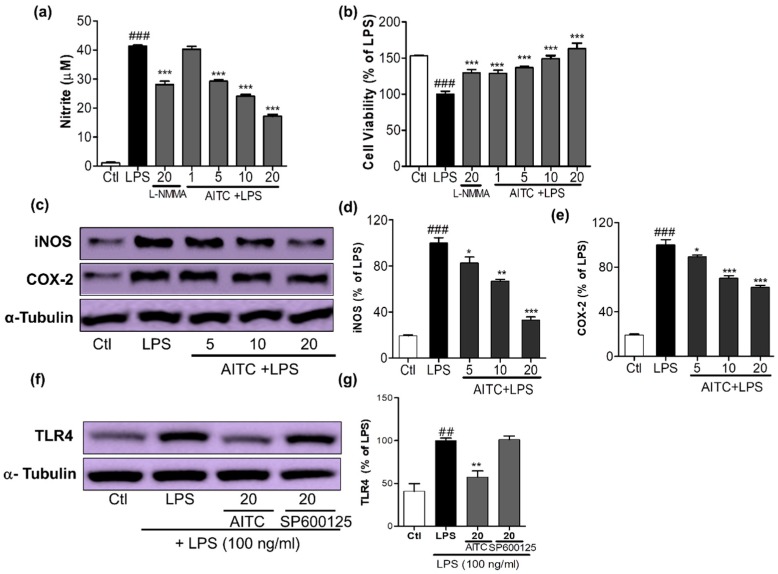
Effects of AITC on NO production, cell viability, inducible nitric oxide synthase (iNOS) and cyclooxygenase-2 (COX-2) expression, and toll like receptor (TLR4) inactivation in LPS-stimulated BV2 cells. BV2 cells were pretreated with various concentrations of AITC (µM) for 30 min before treatment with 100 ng/mL LPS. After LPS activation, 24 h of incubation was performed for the nitrite measurement and cell viability assay, 6 h of incubation was performed for the iNOS and COX-2 expression, and 10 min of incubation was performed for the TLR4 inactivation measurement via Western blotting. The MTT assay was used to measure the cell viability, and Griess reagents were used to measure the NO level. (**a**) NO production; (**b**) Cell viability of BV2 microglia after treatment with compounds with or without LPS. *N*-Monomethyl-l-arginine (NMMA; 20 µM) was used as the positive control; (**c**) AITC reduced the LPS-induced expression of iNOS and COX-2 in BV2 cells; (**d**,**e**) Quantitative band intensity of iNOS and COX-2 expression. Band intensity is expressed as the percentage of the LPS-treated group (set as 100%); (**f**) AITC significantly reduced the activation of TLR4 against LPS; (**g**) Quantitative band intensity of TLR4 expression. α-Tubulin was used as the loading Ctl. LPS-treated group was considered as 100% for densitometric analysis. All data are presented as the mean ± SEM of three independent experiments performed with *n* = 3. * *p* < 0.05, ** *p* < 0.01, and *** *p* < 0.001 vs. LPS-treated group; and ## *p* < 0.01, ### *p* < 0.001 vs. untreated Ctl group. Ctl: control; µM concentration was used.

AITC and SA were screened for their ability to inhibit NO production and their cell viability against LPS-activated BV2 (murine microglia) cells; together with its extract wasabi, AITC was found to be more potent without cellular toxicity (Figure 1). It not only reduced toll like receptor (TLR4) activation, but also decreased LPS-induced NO production in BV2 murine microglial cells in a concentration-dependent manner. AITC reduced NO levels following LPS stimulation from 41.39 ± 0.30 µM in LPS, to 40.28 ± 1.00 µM, 29.29 ± 0.55 µM, 24.16 ± 0.52 µM, and 17.22 ± 0.58 µM at concentrations of 1, 5, 10, and 20 µM, respectively (Figure 2a). The inhibitory effect of AITC was more potent than that of the well-known inducible nitric oxide synthase (iNOS) inhibitor *N*-Monomethyl-l-arginine (NMMA; 28.13 ± 1.26 µM at 20 µM) [21] at doses of 10 and 20 µM. Besides reducing NO production, AITC protected against LPS-induced cell activation and death in a concentration-dependent manner (Figure 2b). AITC reduced the expression of iNOS and cyclooxygenase-2 (COX-2) in a concentration-dependent manner, suggesting that it had significant anti-inflammatory properties in LPS-stimulated BV2 cells (Figure 2c–e). Being a TLR4 agonist, LPS can activate the receptor and initiate the inflammatory cascade by activating MAPK signaling, the nuclear factor (NF-κB-mediated transcription), and further production of iNOS, NO, COX-2, PGE-2, and proinflammatory cytokines. Hence, we investigated the expression of TLR4 immediately after LPS activation in BV2 cells pretreated with AITC. TLR4 significantly reduced the activation after treatment with 10 µM of AITC, but not with the c-Jun N-terminal kinase (JNK) inhibitor SP600125 (Figure 2f,g).

### 2.2. Effects of AITC on LPS-Induced MAPK Signaling in BV2 Cells

**Figure 3:**
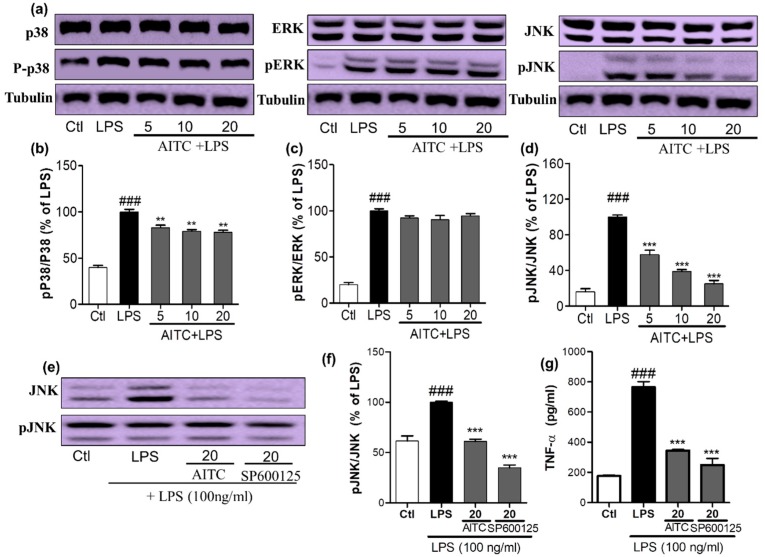
Effects of AITC on LPS-induced mitogen activated protein kinase (MAPK) signaling in BV2 cells. BV2 cells were pretreated with or without AITC for 30 min before LPS stimulation. Then, LPS-activated cells were incubated for 30 min. (**a**) AITC altered the phosphorylation of the MAPKs (p38, c-Jun N-terminal kinase (JNK), and extracellular signal-related kinase—ERK), which was determined by Western blot analysis; (**b**–**d**) Band intensities for p-p38/p38, pERK/ERK, and pJNK/JNK as percentages of the LPS-treated group (set as 100%), respectively; (**e**) Comparison of pJNK inhibitory activity between AITC and SP600125 by Western blot analysis; (**f**) Quantitative band intensity of pJNK/JNK expression; (**g**) Comparison of tumor necrosis factor-α (TNF-α) inhibitory activity between AITC and SP600125. α-Tubulin was used as the loading Ctl. LPS treated group was considered as 100% for densitometric analysis. All data are presented as the mean ± SEM of three independent experiments performed, with *n* = 3. ** *p* < 0.01, and *** *p* < 0.001 vs. LPS-treated group; and ### *p* < 0.001 vs. Ctl group. Ctl: control; µM concentration was used.

To confirm the anti-inflammatory properties of AITC, we investigated the effects of AITC on the production of MAPK family proteins. We assessed the phosphorylation of p38, extracellular signal-related kinase (ERK), and JNK by a Western blot analysis using antibodies against the phosphorylated and total forms the proteins. BV2 cells were pretreated with different concentrations of AITC, and stimulated with LPS for 30 min. AITC dose-dependently decreased the p38 phosphorylation. However, it did not significantly alter the ERK phosphorylation, but did considerably reduce the JNK phosphorylation (Figure 3a–d). The inhibition of JNK phosphorylation by AITC was later compared with the effect of the well-known JNK inhibitor SP600125. AITC showed a similar effect to the JNK inhibitor in reducing JNK phosphorylation (Figure 3e,f). These results suggest that the MAPK-mediated anti-neuroinflammatory and neuroprotective effects of AITC may involve the JNK pathway. Reports suggested that JNK-mediated inflammation is directly related to the induction of neuronal death as well as the neurodegenerative condition, through enhanced TNF-α/NF-κB signaling, or vice versa [22]. In addition, TNF-α mediates the activation of NF-κB and JNK signaling cascades in retinal ganglion cell death [23]. This cross-relation between TNF-α, NF-κB, and JNK demonstrated molecular mechanisms of AITC in the prevention and treatment of neuroinflammation and neurodegeneration in the brain. Therefore, we compared the inhibitory effects of AITC and JNK on TNF-α production in LPS-activated BV2 cells. AITC showed similar results as SP600125, even in the inhibition of TNF-α production (Figure 3g). Hence, AITC modulated MAPKs, and reduced neuroinflammation and neurodegeneration caused by the LPS treatment in murine microglial cells.

### 2.3. Effects of AITC on LPS-Induced NF-κB Activation in Murine Microglial Cells

**Figure 4:**
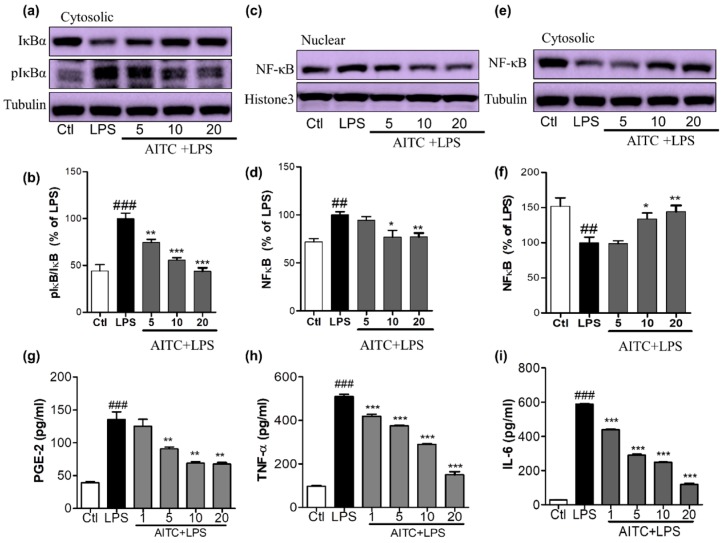
Effects of AITC on LPS-induced nuclear factor (NF)-κB activation and proinflammatory cytokine production in BV2 cells. BV2 cells were treated with AITC 30 min prior to LPS treatment and incubated. After 1 h of LPS treatment, treated cells were prepared to separate nucleolar and cytosolic fractions using a nuclear extraction kit. The expression of nucleolar and cytosolic NF-κB, cytosolic IκB, and p-IκB was measured via Western blotting. (**a**,**b**) Cytosolic IκB and p-IκB expression and its densitometric analysis; (**c**,**d**) Nucleolar NF-κB and its densitometric analysis; (**e**,**f**) Cytosolic NF-κB and its densitometric analysis. The LPS-treated group was considered as 100%. α-Tubulin was used as the loading Ctl for cytosolic protein, while histone-3 was used as the loading Ctl for nucleolar fractions. AITC-treated (24 h) and LPS-activated BV2 cell supernatant was collected and an ELISA was performed to measure prostaglandin E2 (PGE-2), TNF-α, and interleukin-6 (IL-6) production; (**g**–**i**) Measurement of PGE-2, TNF-α, and IL-6 production in LPS-activated BV2 cells treated with or without AITC. Data are presented as the mean ± SEM of three independent experiments performed, with *n* = 3. * *p* < 0.5, ** *p* < 0.01, and *** *p* < 0.001 vs. LPS-treated group; and ## *p* < 0.01, ### *p* < 0.001 vs. Ctl group. Ctl: control; µM concentration was used.

NF-κB is an important upstream modulator of the proinflammatory cytokines iNOS and COX-2 and other inflammatory mediators. LPS significantly enhanced the DNA-binding activity of NF-κB in microglia [24]. The increase of nucleolar NF-κB was significantly ameliorated by pretreating cells with 5, 10, and 20 µM of AITC, as shown in Figure 4a,b. NF-κB inactivation can be mediated by reduced IκB phosphorylation. This inactivated NF-κB with the treatment of AITC can be clearly seen with the increased expression of cytosolic NF-κB with AITC treatment, as shown in Figure 4c,d. To further investigate whether AITC inhibited LPS-induced phosphorylation and subsequent degradation of IκB [24], the cytosolic expression of IκB and p-IκB was observed. Pretreatment of BV2 cells with AITC decreased IκB phosphorylation in response to the LPS, which indicated that the subsequent NF-κB inactivation was induced by AITC in a dose-dependent manner (Figure 4e,f).

### 2.4. Effects of AITC on PGE-2, TNF-α, and IL-6 Production in LPS-Stimulated BV2 Cells

NO production triggers multiple downstream effects that are part of the inflammatory cascade. Activated microglia increases the production and release of prostaglandins and proinflammatory cytokines, such as TNF-α and IL-6, in the affected area. To elucidate the inhibitory effect of AITC on LPS-stimulated PGE-2, TNF-α, and IL-6 production, we performed an ELISA of BV2 cell supernatant following 24 h of treatment. We found that AITC inhibited LPS-stimulated production of PGE-2, TNF-α, and IL-6 in activated microglia in a promising concentration-dependent manner. AITC reduced the PGE-2 release in BV2-conditioned media from 135.60 ± 11.45 µM (LPS only) to 125.10 ± 10.55 µM, 90.80 ± 2.79 µM, 69.05 ± 2.33 µM, and 67.43 ± 2.41 µM, at concentrations of 1, 5, 10, and 20 µM, respectively (Figure 4e). The production of proinflammatory cytokines worsens the inflammatory cascade in neuroinflammatory conditions. The inhibition of the proinflammatory cytokines produced by activated microglia is necessary for neuroprotection. Without LPS stimulation, the TNF-α level in media from BV2 cells was 97.10 ± 3.00 pg/mL; LPS stimulation increased this to 509.90 ± 9.87 pg/mL, and it was reduced to 419.60 ± 8.43 pg/mL, 375.3 ± 4.18 pg/mL, 289.7 ± 3.47 pg/mL, and 150.0 ± 14.61 pg/mL following treatment with AITC at 1, 5, 10, and 20 µM, respectively (Figure 4f). Similarly, LPS increased the IL-6 production to 588.00 ± 4.55 pg/mL from 28.58 ± 067 pg/mL in the control group. Later, this was reduced to 439.4 ± 4.16 pg/mL, 290.20 ± 7.33 pg/mL, 248 ± 2.08 pg/mL, and 119.80 ± 4.77 pg/mL with AITC pretreatments of 1, 5, 10, and 20 µM, respectively (Figure 4g). Thus, AITC drastically reduced the release the proinflammatory cytokines TNF-α and IL-6 in LPS-activated BV2 cells, which indicates that it has potent anti-inflammatory potential.

### 2.5. Effects of AITC on Activated Microglia-Induced Neurotoxicity in N2a Cells

**Figure 5:**
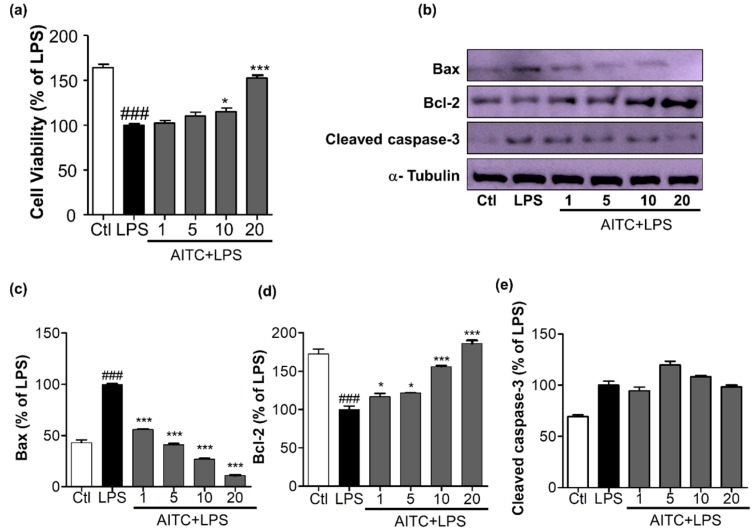
Effects of AITC on activated microglia-induced neurotoxicity in mouse neuroblastoma (N2a) cells. BV2 microglial cells were pretreated with 1, 5, 10, or 20 µM of AITC for 30 min, and stimulated with LPS (100 ng/mL) for the next 24 h. After 24 h of LPS treatment, the culture medium was collected and transferred to dishes plated with N2a cells, and incubated for another 24 h. Later, cell viability of the N2a cells was analyzed, and cell lysate was prepared from the same N2a cells. (**a**) Cell viability was measured using the MTT assay; (**b**) Cell lysates were prepared to evaluate protein levels of apoptosis-related factors. The expression of cleaved caspase-3, Bax, and Bcl-2 were evaluated via Western blot analyses; (**c**–**e**) Quantitative band intensity calculation for Bax, Bcl-2 (B-cell lymphoma 2), and cleaved caspase-3, respectively. α-Tubulin was used as the loading Ctl. Data represent the mean ± SEM of three independent experiments performed, with *n* = 3. * *p* < 0.5 and *** *p* < 0.001 vs. LPS-treated group; and ### *p* < 0.001 vs. untreated Ctl group. LPS-treated group was considered as 100%. Ctl: control; µM concentration was used.

The production of NO, COX-2, iNOS, PGE-2, and proinflammatory cytokines leads to neuronal death, which is mediated by increased levels of apoptotic proteins, such as Bax and cleaved caspase-3, and decreased levels of anti-apoptotic proteins, such as Bcl-2. We determined the effects of AITC on apoptosis in neuronal cells. We treated LPS-activated microglia with or without AITC for 24 h; late conditioned media were transferred from activated BV2 cells to neuronal cells for the next 24 h. AITC significantly increased neuronal cell viability dose-dependently. LPS reduced neuronal cell viability from 163.90 ± 3.93% (control group) to 100.00 ± 1.60% (LPS-only group); AITC protected cells from LPS-induced death. Cell viability increased to 102.20 ± 2.81%, 110.10 ± 4.47%, 115.20 ± 3.91%, and 152.50 ± 3.06% with AITC treatments of 1, 5, 10, and 20 µM, respectively (Figure 5a). AITC decreased the expression of Bax and cleaved caspase-3 in a dose-dependent manner. AITC increased the expression of the anti-apoptotic protein Bcl-2 in neurons in a concentration-dependent manner (Figure 5b–e). Taken together, these results suggested that the reduced expression of Bax and cleaved caspase-3 and increased production of Bcl-2 in neuroblastoma cells were the mechanisms underlying the protective effect of AITC against LPS-induced neuronal death. These results explain the neuroprotective effects of AITC against LPS-activated neuroinflammation.

### 2.6. Effects of AITC on Neurite Morphology and Neurite Outgrowth in N2a Cells

**Figure 6:**
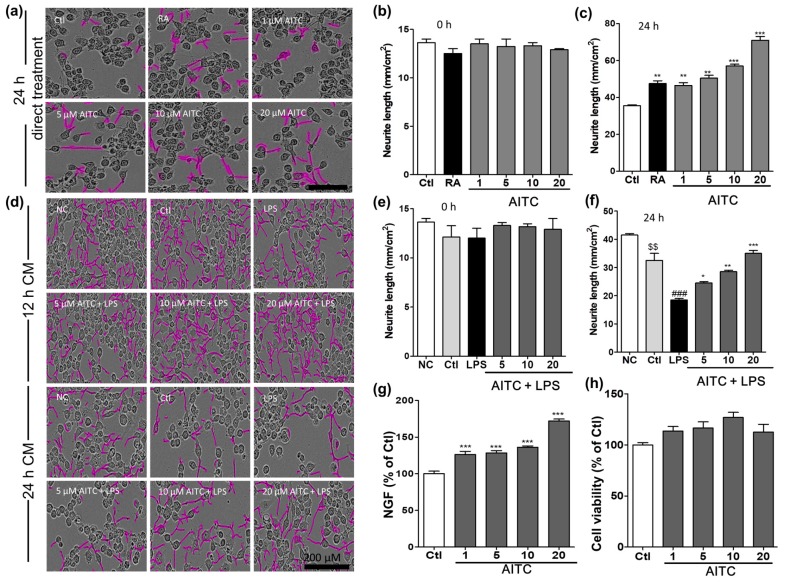
Effects of AITC on neurite morphology and neurite outgrowth in N2a cells and nerve growth factor (NGF) production and viability in C6 glioma cells. Neurite length in N2a cells was measured at regular intervals over 24 h after AITC treatment (1, 5, 10, 20 µM), and images of the cells were captured at the end of 24 h. (**a**) Neuronal cell morphology after treatment with AITC only. Neurite outgrowth is shown in pink; (**b**) Neurite length before AITC treatment; (**c**) Neurite length after 24 h of AITC treatment. Retinoic acid (10 µM) treatment was used as the positive Ctl. BV2 cells were treated with AITC 30 min prior to LPS activation, and incubated for 24 h with AITC and LPS treatment. The next day, the CM of AITC- and LPS-treated BV2 cells was transferred to N2a cells and incubated to IncuCyte immediately. Neurite lengths as neurite outgrowth pictures were measured at every 2 h interval starting from 0 h; (**d**) N2a cell morphology after activated BV2 CM treatment; (**e**) Neurite length at 0 h of CM treatment; (**f**) Neurite length after 24 h of CM treatment. C6 cells were treated with AITC for 24 h, the CM was collected for the ELISA of NGF production, and the cell viability assay was performed in the treated cells; (**g**) NGF production in C6 glioma cell as representative cells of astrocyte; (**h**) Viability of C6 cells after AITC treatment. The data shown represent the mean ± SEM of three independent experiments performed, with *n* = 3. * *p* < 0.5, ** *p* < 0.01 and *** *p* < 0.001 vs. LPS-treated group, $$ *p* < 0.01 vs. NC group, and ### *p* < 0.001 vs. Ctl CM group. Ctl: control, RA: Retinoic acid; NC: normal control, and CM: conditioned medium; µM concentration was used.

Mouse neuroblastoma (N2a) cells were treated with different concentrations of AITC and retinoic acid, and neurite outgrowth was evaluated. The N2a cells ceased to proliferate and began to differentiate, as shown by neurite outgrowth, in response to serum starvation, retinoic acid, or growth factors such as neurotrophins and glial cell-derived neurotrophic factor family ligands. The neurite length was measured using the IncuCyte imaging system for up to 24 h after AITC treatment. AITC dose-dependently increased the neurite length compared with untreated cells. Initially, the neurite length in the two groups was similar (100%); however, after 24 h of AITC treatment, significant changes occurred in the morphology of neurites, and neurite outgrowth increased in the neuroblastoma cell line (Figure 6a). The neurite length after 24 h of AITC treatment was significantly increased in a concentration-dependent manner at 1, 5, 10, and 20 µM, respectively. AITC was more effective at increasing neurite length than the positive control retinoic acid (123.0 ± 0.43% at 10 µM), even at lower concentrations (Figure 6b,c). In addition, we investigated the role of AITC in increasing neurite growth even after the LPS-activated and AITC-treated BV2 microglial conditioned medium was transferred to the N2a cells. AITC not only increased the viability of N2a cells, but also induced neurite growth against activated microglia-induced toxicity (Figure 6d–f). These results validate the neuroprotective, as well as anti-neuroinflammatory, effects of AITC.

### 2.7. Effects of AITC on NGF Production and Viability in C6 Cells

Activated microglia produce various inflammatory mediators as well as proinflammatory cytokines that activate astrocytes in a positive feedback loop. To protect the neuronal environment from such toxins, neurons and astrocytes produce neurotrophins such as nerve growth factor (NGF), brain derived neurotrophic factor (BDNF), and neurotrophin (NT3). Hence, compounds that enhance the production of neurotrophins can protect cells and the system as a whole from the harmful effects of activated microglia. To measure the NGF content in the cell culture medium, C6 cells were seeded onto 24-well plates and treated with different concentrations of AITC. The NGF concentration in the culture medium was determined using ELISA, and a Western blot analysis was performed to confirm the NGF protein expression. AITC significantly increased the levels of NGF in the medium (Figure 6g) in a concentration-dependent manner. The percentages of NGF after astrocytes were treated with AITC (compared with untreated cells) was 126.3 ± 4.30%, 128 ± 3.19%, 136.2 ± 1.67%, and 172.00 ± 2.66% at concentrations of 1, 5, 10, and 20 µM, respectively. Furthermore, AITC is not cytotoxic to C6 cells (Figure 6h). This provided a clue about the neuroprotective mechanism of AITC under inflammatory conditions or in neurodegeneration.

## 3. Discussion

*W. japonica* has various therapeutic effects, such as anti-obesity, antioxidant, and anti-hypercholesterolemic effects, which have been previously reported [12]. The anti-inflammatory and strong anticancer potential of 6-methylsulfinylhexyl isothiocyanate obtained from *W. japonica* has increased the scientific interest in this plant [25,26,27]. The presence of several phenolic compounds, AITC, and SA make this plant quite promising for the treatment of various diseases [17,18] Our group previously standardized the presence of SA and AITC in a wasabi extract. This showed that the presence of significant amounts of SA and AITC in the wasabi extract might have played an important role in the amelioration of irritable bowel syndrome-like symptoms in a zymosan-induced mouse model [28]. We also tried to understand the anti-inflammatory effect of the same extract and its representative compounds, SA and AITC, in an LPS-activated, microglia-mediated neuroinflammation model. Originally, an anti-inflammatory effect of SA was observed at 40–160 µM in RAW 264.7 macrophages [29]. However, the anti-inflammatory effects of AITC in this experiment were observed at 1–20 µM, which indicated that AITC is more potent and pharmacologically active against LPS-induced neuroinflammation than SA is. Previous studies reported that the *W. japonica* extract containing AITC may inhibit LPS-induced inflammation in RAW 264.7 macrophages; however, the molecular mechanism was not reported in detail [10,12]. In this study, we attempted to identify the exact target of AITC to demonstrate its anti-neuroinflammatory activity against LPS-induced injury to brain cells. Although it is known to be involved in the regulation of the NF-κB-mediated inflammatory cascade in cancer pathology [17], the exact role of LPS-mediated neuroinflammation in murine microglia has not yet been reported. Thus, we hypothesized that AITC may regulate neuroinflammation by inhibiting microglial activation and associated CNS toxicity.

LPS is a TLR4 agonist that activates microglia and alters the levels of MAPKs, NF-κB, iNOS, COX-2, PGE-2, proinflammatory cytokines, nitrites, and reactive oxygen species [24,30], which are involved in various neurodegenerative diseases such as AD, PD, and multiple sclerosis (MS) [31,32]. Pretreatment of macrophages with 1–20 μM of AITC significantly reduced the TLR4 receptor activation and produced such inflammatory mediators in a concentration-dependent manner. Transcription of such inflammatory mediators occurs following the activation of TLR4, and involves MAPK family member (P38, ERK, and JNK) signaling, resulting in NF-κB-mediated transcription [33,34]. Interestingly, the suppressive effects of AITC on TNF-α and IL-6 production were greater than those on PGE-2, iNOS, and COX-2. Furthermore, AITC strongly inhibited LPS-induced JNK and p38 phosphorylation, but did not alter ERK phosphorylation, which supports the notion that AITC has a potent inhibitory effect on the production of proinflammatory cytokines, such as TNF-α, through JNK-NF-κB signaling. ERK was previously reported to serve as an anti-inflammatory signal that suppresses the expression of NF-κB-dependent inflammatory gene*s* by inhibiting I-κB kinase activity [35]. On the contrary, JNK is an important stress-responsive kinase implicated in neuroinflammation, blood brain barrier (BBB) disruption, and oligodendroglial apoptosis following LPS-induced microglial activation in vitro [36]. LPS-activated microglia express TNF-α and JNK, which together precede cell death through inflammation and apoptosis; therefore, the inhibition of TNF-α- TNF-α receptor (TNFR1) or JNK signaling exerts neuroprotective effects [37]. The JNK and TNF-α inhibitory potential of AITC was found to be almost similar to the well-known JNK inhibitor compound SP600125. Reports suggested that JNK-mediated inflammation is directly related to the induction of neuronal death and neurodegenerative conditions through enhanced TNF-α/NF-κB signaling, or vice versa [22]. Furthermore, TNF-α mediates the activation of NF-κB and JNK signaling cascades in retinal ganglion cell death [23]. This cross-relation between TNF-α, NF-κB, and JNK demonstrated the molecular mechanism underlying the prevention and treatment of neuroinflammation and neurodegeneration in the brain by AITC. Therefore, we compared the effects of AITC and the JNK inhibitor on TNF-α production in LPS-activated BV2 cells. AITC showed similar results as SP600125, even in the inhibition of TNF-α production. The inhibition of JNK phosphorylation and TNF-α production was confirmed using NF-κB and its proteins. NF-κB is a major transcription factor responsible for the production of inflammatory mediators, as well as proinflammatory cytokines, and it is particularly controlled by MAPK signaling. The AITC not only inhibited the phosphorylation of the MAPK family proteins, but also decreased the expression of the nuclear NF-κB, decreased the cytosolic p-IκB expression, and reduced IκB degradation. In addition, AITC also reduced the release the proinflammatory cytokines. We proved its role in the sequence of inhibition of MAPK-NF-κB-proinflammatory cytokine cascades. In this study, we clearly showed the effects of AITC on NF-kB activation in both nuclear and cytosolic fractions, and we found that AITC concentration-dependently reduced the activated NF-kB level in the nucleolar fractions, whereas it increased the degradation of NF-kB in the cytosolic fractions. Furthermore, AITC dose-dependently reduced the production of TNF-α and IL-6, and considerably inhibited JNK phosphorylation, which was followed by a reduction of NF-κB-mediated transcription. We confirmed that the AITC-mediated anti-neuroinflammation occurs through the inhibition of JNK/NF-κB/TNF-α signaling and their inflammatory cascades in LPS-activated BV2 cells. AITC inhibited the phosphorylation of IκB and the expression of NF-κB significantly, compared with the LPS treatment, in a concentration-dependent manner. Furthermore, AITC dose-dependently reduced the production of TNF-α and IL-6, and considerably inhibited JNK phosphorylation, which was followed by a reduction of NF-κB-mediated transcription. This confirmed that the AITC-mediated anti-neuroinflammation occurred through JNK/NF-κB/TNF-α molecules and the control of their mechanism. From these results, we hypothesized that the promising effects of AITC in controlling these molecules may also be involved in neuroprotection. Decreased cross-talk between TNF-α and JNK, which is involved in the preventative mechanism of AITC in LPS-induced neuroinflammation, may be a key target for the prevention and treatment of neurodegeneration and neurotoxic injury. This result suggests that the regulation of p38 and JNK by AITC plays an important role in the production of inflammatory cytokines in LPS-induced conditions.

Activated microglia-derived TNF-α, NF-κB, and JNK work together with the Bcl-2 family protein Bax in neurons to respond to CNS insults that promote the release of factors such as cytochrome-c, which promote caspase activation and ultimately cause apoptotic cell death in neurons [38]. Immune cells present in the brain control the balance between pro- and anti-apoptotic factors through the Bcl-2 family [39]. The expression of pro-apoptotic proteins such as cleaved caspase-3 and Bax is significantly increased, while that of the anti-apoptotic protein Bcl-2 is decreased during neuronal apoptosis [39], which ultimately causes the loss of neurons or axons characteristic of neuronal degeneration [40]. Previous studies reported that an increase in the cleaved caspase-3 expression is a hallmark of apoptosis [41]. In our study, AITC not only reduced activated microglia-induced toxicity in N2a cells by increasing cell viability, but also induced the expression of the anti-apoptotic protein Bcl-2, and reduced the expression of the pro-apoptotic proteins Bax and cleaved caspase-3 in neuronal cells subject to activated microglia-induced toxicity. Additionally, neurite outgrowth, which was mediated through the neurotrophic factor, led to neuroprotection by preventing the endotoxin-induced neurodegeneration. Furthermore, significant increases in the neurite length and density after AITC treatment relative to retinoic acid treatment in N2a cells was confirmed with the increased neurite length in response to LPS-activated, microglia-induced toxicity to neurons. LPS-treated conditioned medium showed the degradation of neurite outgrowth and cell death; a reversed and increased neurite length was observed in the AITC-treated group. Moreover, AITC-mediated inhibition of JNK phosphorylation followed by the subsequent downregulation of TNF-α and NF-κB may be responsible for the protection of neurons against activated microglia-induced toxicity. Furthermore, AITC treatment increased the NGF production in astrocytes, which helps in protecting neurons against neurodegenerative conditions. This demonstrated the strong neuroprotective efficacy of AITC. NGF is secreted by astrocytes and can ameliorate inflammation-induced damage to neurons, and prevent apoptotic neuronal cell death [42]. Therefore, AITC not only inhibits activated microglia-induced neuroinflammation and apoptosis, but also protects neurons from CNS insult through neurite outgrowth and expression of neurotrophins such as NGF. These results collectively suggest that the increase of NGF production by AITC might further support neurons to survive, as well as protect neurite outgrowth against various brain insults. These in vitro results can provide a strong clue for the evaluation of the biological role of AITC against various neuroinflammatory as well as neurodegenerative conditions in vivo.

AITC reduced the production of inflammatory mediators and proinflammatory cytokines, and decreased NF-κB-mediated transcription. An additional effect of AITC inhibiting JNK and I-κB phosphorylation following LPS-activated, microglia-induced neuroinflammation in BV2 cells further demonstrated its neuroprotective activity. Furthermore, the strong anti-apoptotic efficacy of AITC protected neuronal cells from activated microglia-induced toxicity. This concluded that JNK/NF-κB/TNF-α-mediated cross-talk in the activated microglia is the major underlying reason for neuronal death through the activation of the *Bcl-2* gene family in various neurodegenerative diseases. AITC played an important role here by inhibiting the activation of JNK, NF-κB, and TNF-α production in LPS-activated microglia, which not only reduced microglia activation, but also prevented neuronal death by decreasing pro-apoptotic proteins and inducing anti-apoptotic proteins. The enhanced neurite outgrowth observed in neuronal cells and the increased NGF production in astrocytes further supported the neuroprotective effect of AITC in neuroinflammatory as well as neurodegenerative conditions. The limitations of this study were that we used a BV2 murine microglial cell line in order to study the anti-inflammatory potential of AITC that might not have simulated the actual in vivo inflammatory conditions, such as CNS disorders; therefore, further study to investigate the role of AITC in neuroinflammatory disorders, in particular those involving microglia activation, might elaborate on the actual role of AITC in the therapeutic paradigm. Our study, however, provides the basis for such future investigations, which might further ensure the possible use of AITC as a promising nutraceutical and in functional foods, for the regulation of neuroinflammation and neurodegenerative conditions.

## 4. Materials and Methods

### 4.1. Materials

Dulbecco’s modified Eagle medium (DMEM), fetal bovine serum (FBS), and penicillin-streptomycin (PS) were purchased from Invitrogen (Carlsbad, CA, USA). LPS, *N*-Monomethyl-l-arginine (NMMA), and AITC were purchased from Sigma-Aldrich (St. Louis, MO, USA). ELISA kits for TNF-α, IL-6 and NGF were bought from R&D Systems (Minneapolis, MN, USA), PGE-2 kit was purchased from cayman chemical (Ann Arbor, MI, USA). Primary and secondary antibodies against iNOS, COX2, ERK, pERK, JNK, pJNK, p38, pP38, Bax, Bcl-2, cleaved caspase-3, and tubulin were purchased from Cell Signaling (Beverly, MA, USA). All other chemicals and reagents were purchased from Sigma Chemical Co. (St. Louis, MO, USA).

### 4.2. Wasabi Extraction

*Wasabi koreana* was purchased from Semtong Farm (Cheorlwon, Korea). It was dried properly and grinded into a fine powder. Extraction of wasabi was performed according to the protocol described by Park et al. [28], with slight modification. About 100 g of wasabi powder was extracted with 50% ethanol under sonication for about 1 h. The extract was filtered and evaporated using a rotary evaporator. For the complete lyophilization of the extract, evaporated extract yield was further dried in a freeze drying system. We obtained about 9.5% dried yield extract. The extract was stored in a refrigerator and dissolved in distilled water to make 100 mg/mL stock solution for the treatment purpose. The standardization of the wasabi extract, SA and AITC was previously reported by our group [28].

### 4.3. Cell Culture

In this study, three different CNS cell lines, BV2 murine microglia, C6 glioma, and N2a mouse neuroblastoma cells, were used to study the anti-neuroinflammatory and neuroprotective effects of AITC. The BV2 microglial cell line was obtained as a gift from E. Choi of Korea University (Seoul, Korea), C6 glioma cells were purchased from the Korean Cell Line Bank (Seoul, Korea), and N2a cells were originally obtained from the American Type Culture Collection (Manassas, VA, USA). All cell lines were maintained in DMEM, supplemented with 10% heat-inactivated FBS, penicillin (1 × 10^5^ U/L), and streptomycin (100 mg/L), in a humidified incubator with 5% CO_2_ at 37 °C [43].

### 4.4. Cell Viability Assay

To determine the potential cytotoxic properties of AITC on the different CNS cell lines, the viability was evaluated by measuring the ability of viable cells to reduce yellow-colored MTT (3-(4,5-dimethylthiazol-2-yl)-2,5-diphenyltetrazolium bromide) to purple formazan. Cells were cultured in 96-well plates at a density of 4 × 10^4^ cells/well. MTT was dissolved in DMEM and added to the culture medium at a final concentration of 0.5 mg/mL. After 1 h of incubation, the media was carefully removed, and 200 μL of DMSO was added to each well. The optical density (OD) was measured on a plate reader at 570 nm. Results are expressed as a percentage of the LPS-treated group [44].

### 4.5. Nitric Oxide and Proinflammatory Cytokine Measurement

The NO and proinflammatory cytokines’ inhibitory effects of AITC on LPS-stimulated microglia were studied using BV2 cells. These cells were seeded in a 96-well plate at a density of 4 × 10^4^ cells/well, and were treated with various concentrations of AITC 30 min prior to the LPS (200 ng/mL) treatment. The treated plate was incubated for 24 h. The nitrite level was measured in the culture media using a Griess reagent (1% sulfanilamide in phosphoric acid, and 0.1% *N*-1-napthylethylenediamine dihydrochloride). The supernatant (50 μL) was mixed with an equal volume of the Griess reagent, and the OD was measured at 570 nm. NG-Mono-methyl-l-arginine (l-NMMA), a well-known NOS inhibitor [21], was used as the positive control. ELISA kits were used to measure the levels of proinflammatory cytokines (TNF-α and IL-6), PGE-2, and NGF, according to the manufacturer’s protocol.

### 4.6. NGF Assay

In this study, C6 glioma cells were used to measure NGF release into the culture medium. C6 cells were seeded onto 24-well plates at a density of 1 × 10^5^ cells/well [43]. After 24 h, the cells were treated with DMEM containing 2% FBS and antibiotics with different concentrations of AITC for an additional 24 h. The culture media were harvested, and NGF was measured using an ELISA development kit.

### 4.7. BV2 Conditioned Medium Treatment with N2a Cells

BV2 microglial cells were seeded in a 6-well plate at a density of 4 × 10^4^ cells/well and pretreated with AITC, followed by activation with LPS. After 24 h of AITC and LPS treatment, the conditioned medium of treated cells was transferred to N2a cells seeded in a 6-well plate at a density of 1 × 10^4^ cells/well. N2a cells were incubated with conditioned medium for 24 h, and cell images were simultaneously captured. After 24 h of incubation, the cells were used to measure the activated microglia-induced toxicity to N2a cells [43,44].

### 4.8. Neurite Outgrowth Assay

We used mouse N2a cells derived from a neuroblastoma of mice to measure the neurite outgrowth. N2a cells were seeded onto 6-well plates at a density of 1 × 10^4^ cells/well and treated with AITC for 24 h. N2a cells ceased to proliferate and began to differentiate, as evidenced by the neurite outgrowth, in response to serum starvation, retinoic acid, or growth factors such as neurotrophins and glial cell-derived neurotrophic factor family ligands. N2a cell neurite lengths were measured using an IncuCyte imaging system (Essen Instruments, Ann Arbor, MI, USA) [44].

### 4.9. Western Blot Analysis

Proteins obtained from BV2 cells and N2a cells were used for the Western blot analysis. Protein of each sample (30 μg) was loaded and separated by 10% SDS-PAGE, transferred to nitrocellulose membranes, blocked with 5% skim-milk, and incubated overnight with primary antibodies against tubulin (Sigma-Aldrich, St. Louis, MO, USA, Cat. No.: T5168), iNOS (Bioscience Cat. No.: 610333), COX-2 (Santa Cruz, CA, USA, Cat. No.: sc-1745), ERK (Cell Signaling, Danvers, MA, USA, Cat. No.: 9107s), pERK (Cell Signaling Cat. No.: 5013s), JNK (Cell Signaling Cat. No.: 9252s), pJNK (Cell Signaling Cat. No.: 4671s), p38 (Cell Signaling Cat. No.: 8690s), p38 Cell Signaling Cat. No.: 9211s), cleaved caspase-3 (Cell Signaling Cat. No.: 9661s), Bax (Santa Cruz, CA, USA, Cat. No.: Sc-493), and Bcl-2 (Santa Cruz, CA, USA, Cat. No.: Sc492) at 4 °C. Membranes were then incubated with respective horseradish peroxidase-conjugated secondary antibodies, and protein bands were visualized using an enhanced chemiluminescence reagent using the Chemi DocXRS+ imaging system (Bio-Rad, Hercules, CA, USA). Densitometry analyses of the bands was performed by using Image Master 2D Elite software (version 3.1, Amersham Pharmacia Biotech, Buckinghamshire, UK).

### 4.10. Statistical Analysis

All results are presented as means ± SEM. Significant differences between experimental groups were determined using a one-way ANOVA followed by a Newman-Keuls post hoc test using GraphPad Prism 5 (GraphPad Software Inc., La Jolla, CA, USA). A value of *p* < 0.05 was considered statistically significant. Each experiment was performed in triplicate, with *n* = 3.
